# Esthesioneuroblastoma: Unraveling the Enigma and Emerging Paradigms With a Retrospective Cohort and Review of Literature

**DOI:** 10.7759/cureus.87985

**Published:** 2025-07-15

**Authors:** Narendhar Gokulanathan, Vedamanasa Ikkurthi, Senthamizhan Sundaramoorthy, Madhan Raj

**Affiliations:** 1 Medical Oncology, Apollo Hospitals, Bangalore, IND; 2 Radiation Oncology, Homi Bhabha Cancer Hospital and Research Centre (Tata Memorial Center), Chandigarh, IND; 3 Medical Oncology, Regional Cancer Centre, Trivandrum, IND; 4 Radiation Oncology, Tata Memorial Hospital, Mumbai, IND

**Keywords:** esthesioneuroblastoma, imrt, olfactory neuroblastoma, oncology, radiation, radiotherapy, rare tumors, surgery

## Abstract

Background

Esthesioneuroblastoma (ENB), or olfactory neuroblastoma, is a rare malignancy arising from the olfactory epithelium. Standardized treatment protocols are lacking, and existing knowledge is largely derived from retrospective case series.

Methods

This study was undertaken to evaluate treatment outcomes and toxicities associated with multimodal therapy in a rare malignancy. We retrospectively reviewed the clinical, pathological, radiological, and dosimetric data of patients diagnosed with ENB and treated them at the Regional Cancer Centre, Jawaharlal Institute of Postgraduate Medical Education & Research (JIPMER), between 2015 and 2022. All patients underwent baseline imaging, including contrast-enhanced CT of the head, neck, thorax, and abdomen and MRI of the head and neck. Treatment included combinations of neoadjuvant chemotherapy, craniofacial resection, and intensity-modulated radiotherapy (IMRT). Radiological response assessment and clinical follow-up were performed periodically, with the last follow-up in March 2023.

Results

Around eight patients aged 17-68 years (median: 53 years) were included. Most patients presented with Kadish stage C (n=5), followed by stage D (n=3). T3 disease was predominant (7/8), with nodal involvement in four patients. Histologically, most tumors were Hyams grade 3 (n=7), neuron-specific enolase-positive, with mitotic indices of 7-17/10 high-power field. All patients received IMRT (66-70 Gy); five underwent R0 surgery and adjuvant radiotherapy, while three received radical chemoradiation. Neoadjuvant chemotherapy (cisplatin and etoposide) was administered in seven patients and adjuvant in one. At a median follow-up of 51.5 months, four patients were alive and disease-free, one had locoregional recurrence, and three had died (one due to recurrence, one due to a second malignancy, and one unrelated). Treatment-related toxicities were manageable and generally resolved with supportive care.

Conclusion

This case series highlights the feasibility and efficacy of multimodal therapy incorporating surgery, radiotherapy, and chemotherapy in advanced-stage ENB. With careful staging, aggressive local therapy, systemic treatment, and long-term disease control is achievable. However, larger multicentric studies and molecular diagnostics are necessary to formulate management strategies and refine prognostication in this rare tumor.

## Introduction

Esthesioneuroblastoma (ENB), also known as olfactory neuroblastoma, is a rare neoplasm of the olfactory epithelium, which was first described in 1924. It predominantly presents bimodally in the second and sixth decades of life and sporadically across age groups as well. It has equal gender predilection. It has an incidence of 0.4 per million population, with only around 1200 recorded cases in the literature. Due to its rarity, there is a lack of randomized data to provide a streamlined approach to diagnosis and management, and much of the data on ENB has been obtained from valuable case series. Studies have demonstrated a 5-year overall survival of 70%-81.9% and a 10-year overall survival of 63.7% [1-4]. This study was undertaken to evaluate treatment outcomes and toxicities associated with multimodal therapy in a rare malignancy like ENB.

## Materials and methods

This was a single-institution, retrospective cohort study conducted at the Department of Radiation Oncology, Regional Cancer Centre, Jawaharlal Institute of Postgraduate Medical Education & Research (JIPMER), Puducherry, India. A total of eight patients with histologically confirmed ENB and treated with curative intent between January 2015 and December 2022 were identified and included. Data were collected retrospectively from electronic and paper-based medical records, and consent was obtained at the time of clinical follow-up from each individual subject. The final follow-up was completed in March 2023. IRB approval was obtained for this retrospective study.

Patients

The study included patients with histologically confirmed ENB, patients who received radiotherapy (RT) at our center with curative or adjuvant intent, and patients whose complete clinical, pathological, imaging, treatment, and follow-up records were available.

Patients who had received palliative intent RT, patients with incomplete records, or patients lost to follow-up were excluded from the study.

Pretreatment workup and assessment

All patients underwent complete clinical examination and detailed history-taking, followed by staging workup, which included contrast-enhanced CT (CECT) of the head, neck, thorax, and abdomen for all patients. MRI of the head and neck and dental evaluation were also obtained before radiation treatment planning. Endoscopic evaluation was performed in all cases for diagnostic biopsy and local disease assessment. Nodal status, presence of orbital, intracranial, or nodal extension, was documented as per imaging.

Baseline routine blood investigations, including hemogram and renal and liver function tests, were performed. Weekly assessment of blood parameters during RT and prechemotherapy was documented.

All biopsied specimens were reviewed by in-house pathologists specializing in oncopathology. The following histopathological parameters were included during histopathological reporting: Hyams grading, mitotic index, and rosette patterns. Immunohistochemistry markers tested included neuron-specific enolase (NSE), synaptophysin, CD56, cytokeratins, epithelial membrane antigen, and others as clinically indicated. Tumors were staged using the Kadish classification (stages A-D) and AJCC TNM 8th edition (Maxilla, Ethmoid subsites). Postoperative specimens were assessed for resection margins. Treatment decisions were taken after presentation to a multidisciplinary team (MDT) comprising of radiation oncologists, surgical oncologists, head and neck oncologists, radiologists, and medical oncologists.

Chemotherapy

Neoadjuvant regimen included cisplatin (30 mg/m², days 1 to 3) and etoposide (100 mg/m², days 1 to 3) every three weeks. Response Evaluation Criteria in Solid Tumors (RECIST) 1.1 were used to assess response to chemotherapy after three cycles. Concurrent chemoradiation regimen included cisplatin (100 mg/m ² on days 1, 22, and 43) with appropriate premedication protocols. In patients who did not receive neoadjuvant chemotherapy, three cycles of adjuvant chemotherapy with the same regimen were given.

Surgery

Patients with resectable disease amenable to upfront surgical resection and adequate performance status as determined by MDT underwent craniofacial resection and complete excision of tumor as feasible. Patients who responded to chemotherapy (partial response, complete response, and stable disease) as per RECIST 1.1 criteria on response assessment imaging were taken up for craniofacial resection.

RT

Poor responders (progressive disease as per RECIST 1.1) were planned for radical intent radiation therapy. RT was delivered three to four weeks after chemotherapy or surgery. All patients received RT using intensity-modulated RT (IMRT) technique delivered as 6 MV photons. Indications for adjuvant RT were derived from guidelines for standard non-nasopharyngeal head and neck squamous cell carcinomas (HNSCCs).

Dose schedule for adjuvant RT was 66 Gy in 33 fractions (2 Gy/#) and for radical RT, it was 70 Gy in 35 fractions, as prescribed to the planning target volume (PTV). Dose-volume histogram parameters and dosimetric data were obtained from the Varian Eclipse treatment planning system. Target delineation for radical radiation included gross tumor volume (GTV), defined as tumor delineated through simulation CT fused with MRI images. Clinical target volume (CTV) was defined as GTV + 5 mm margin, along with elective nodal fields. For adjuvant setting, CTV was defined as postoperative tumor cavity, mucosal irregularities, involved margins (if applicable), and ipsilateral involved lymphatic fields (if applicable). PTV was defined as CTV + 3 mm margin.

Follow-up and outcome assessment

Patients underwent clinical assessment monthly for the first six months, then every three to six months for the first two years, and annually thereafter. Clinical examination, nasal endoscopy, and imaging (MRI/CT) were performed every six months or earlier if symptomatic. National Cancer Institute - Common Terminology Criteria for Adverse Events (NCI-CTCAE) version 5 (US National Cancer Institute, Bethesda, Maryland, USA) was used to assess adverse events during the course of RT and chemotherapy.

Descriptive statistics (mean, median, and frequencies) were used to summarize demographics and clinical variables.

## Results

A total of eight patients with ENB were treated at our institution between the ages of 17 and 68 years (median: 53 years). The majority (n=6) had an Eastern Cooperative Oncology Group performance status of 0-1. The cohort included five males and three females. Most patients presented with Kadish stage C (n=5), with the remainder at stage D (n=3). T3 disease was predominant (7/8), with nodal involvement (N1) in four patients, while one patient had intracranial extension. 

Histopathologically, all tumors were NSE-positive, predominantly Hyams grade 3 (n=7), with mitotic indices ranging from 7 to 17/10 high-power field (HPF). Presenting symptoms included nasal and olfactory complaints (100%); ophthalmologic (75%), neurologic (62.5%), and nodal (50%) symptoms; and rare features such as seizures and otologic signs.

**Figure 1:**
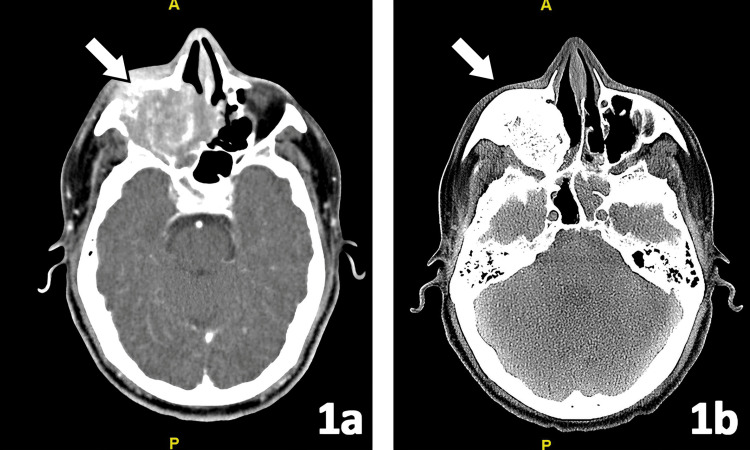
CT transverse section images (a, b) CT images of a patient before and after curative-intent treatment (site of primary lesion shown with an arrow mark)

**Figure 2:**
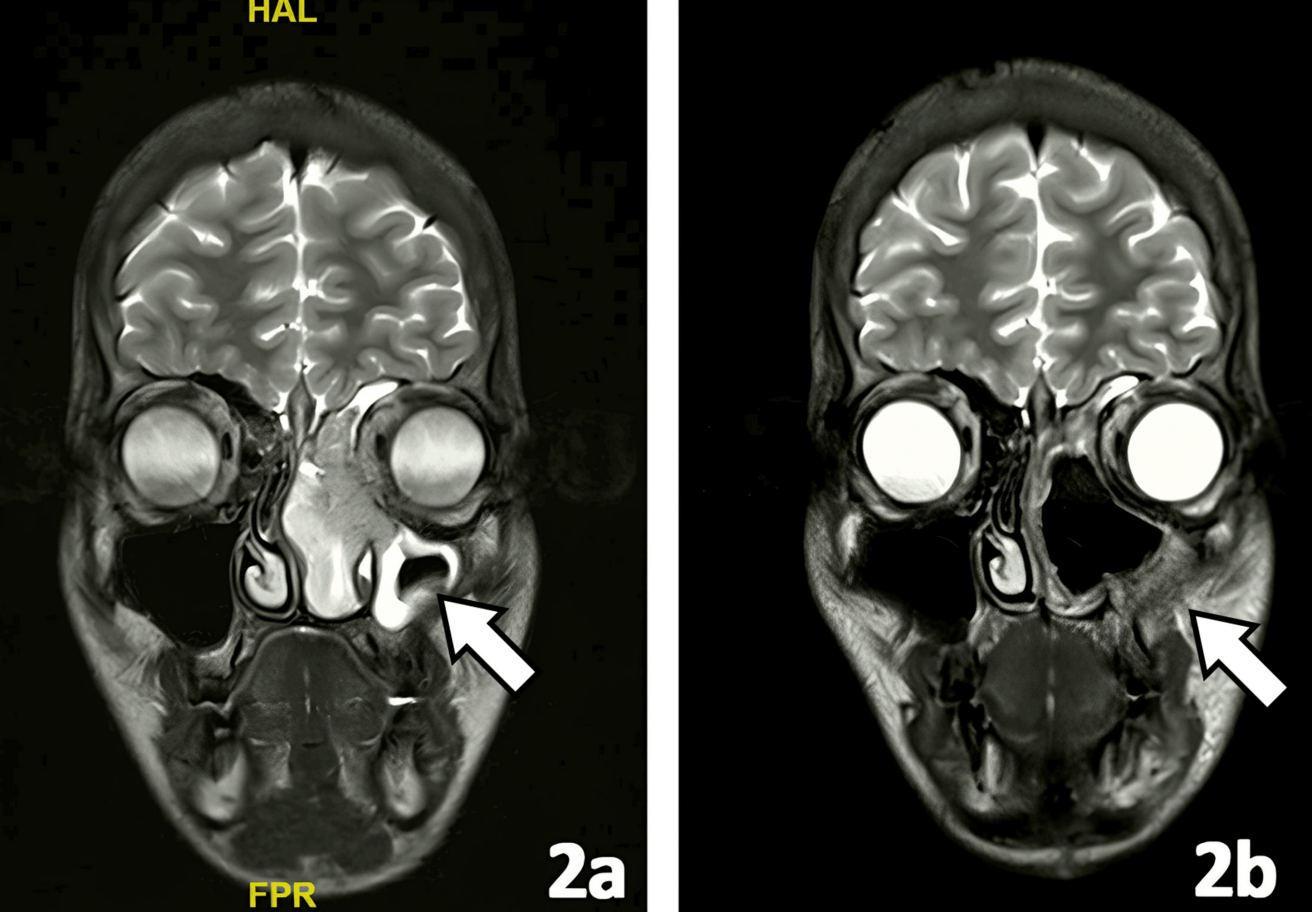
MRI coronal section images (a, b) MRI images of a patient before and after curative-intent treatment (site of primary lesion shown with an arrow mark)

Surgery was performed in five patients with R0 resection. All patients received RT using IMRT: five patients received adjuvant RT at a dose of 66 Gy, while three patients received radical RT at a dose of 70 Gy. Median PTV V95 and CTV V100 were >95% and >97%, respectively. Neoadjuvant chemotherapy with cisplatin and etoposide was delivered in seven patients, and adjuvant chemotherapy in one patient. At a median follow-up of 51.5 months, four patients were alive and disease-free, one had locoregional recurrence, and three had died (due to recurrence, second malignancy, and unrelated cause). The responses to treatment were assessed with CT and MRI, as shown in Figures 1a-2b.

Treatment-related delayed toxicities included postoperative wound infection (n=1), chemotherapy-induced neuropathy (n=3), and radiation-related xerostomia or epiphora (n=3); all resolved with supportive care. The clinical and pathological records are depicted in Tables 1-4 below.

**Table 1:** Patient and treatment characteristics CTV V100: Clinical target volume receiving 100% of the prescribed dose; ECOG: Eastern Cooperative Oncology Group; FU: Follow-up; IMRT: Intensity-modulated radiotherapy; LR: Local recurrence; N: Nodal stage; NED: No evidence of disease; No.: Number; PTV V95: Planning target volume receiving at least 95% of the prescribed dose; RT: Radiotherapy; T: Tumor stage

Patient no.	Age in years	Sex	ECOG	T stage	N stage	Kadish stage	Surgery	RT intent, dose (IMRT)	PTV V95/CTV V100	Chemo intent, cycles (cisplatin and etoposide)	FU (months)	Status at last FU
1	17	M	0	T3	N1	C	Complete excision (R0)	Adjuvant, 66 Gy	97.8/99.3	Neoadjuvant x 2, adjuvant x 1	28	NED
2	59	F	1	T3	N0	C	Complete excision (R0)	Adjuvant, 66 Gy	98.6/99.7	Neoadjuvant x 3	102	NED
3	68	M	1	T4	N0	C	Complete excision (R0)	Adjuvant, 66 Gy	94.5/98.3	Neoadjuvant x 4	39	Expired, LR
4	60	M	1	T3	N1	D	Complete excision (R0)	Adjuvant, 66 Gy	95.9/97.8	Neoadjuvant x 3	64	Expired, due to non-cancer causes
5	22	M	0	T3	N0	C	Complete excision (R0)	Adjuvant, 66 Gy	99.3/99.9	Adjuvant x 3	87	Expired, 2nd malignancy – brainstem glioma
6	50	M	1	T3	N0	C	Not done	Radical, 70 Gy	98.2/99.1	Neoadjuvant x 3	24	NED
7	44	F	2	T3	N1	D	Not done	Radical, 70 Gy	95.5/97.1	Neoadjuvant x 3	75	LR
8	56	N	2	T3	N1	D	Not done	Radical, 70 Gy	98.1/99.2	Neoadjuvant x 3	31	NED

**Table 2:** Pathological characteristics CK: Cytokeratin; EMA: Epithelial membrane antigen; HPF: High-power field; NSE: Neuron-specific enolase

Patient number	NSE	Synaptophysin	CD56	CK	EMA	Additional markers	Rosette type	Necrosis	Mitotic index (per 10 HPF)	Hyams grade
1	+	+	+	+	+	-	Perivascular pseudorosette	No	8	3
2	+	-	+	+	+	-	Homer Wright	No	11	3
3	+	-	-	-	+	Negative for calretinin and CDX2	Homer Wright	No	14	4
4	+	-	+	+	+	-	Homer Wright	No	9	3
5	+	-	+	+	+	-	Flexner Wintersteiner	Yes	16	3
6	+	-	+	+	+	Negative for p63, Cd99 and FLI-1	Perivascular pseudorosette	No	7	3
7	+	+	+	+	+	-	Flexner Wintersteiner	Yes	15	3
8	_+_	+	+	+	+	-	Flexner Wintersteiner	Yes	17	3

**Table 3:** Presenting complaints CN: Cranial nerve; EOM: Extraocular muscle

Presenting complaints	Number of patients
Olfactory – loss of smell, nasal block, epistaxis, and mass descending from the nare	8
Ophthalmologic – proptosis, conjunctival congestion, chemosis, restricted movement of EOM, diplopia, visual dysfunction, and loss of visual acuity	6
Neurologic – CN VII palsy, headache, and facial pain	5
Neck mass (nodal)	4
Intracranial features – seizures	1
Otologic – serous otitis media	1

**Table 4:** After treatment complications during follow-up

After treatment complications	Number of patients (current status)
Surgical – postoperative wound infection	1 (resolved)
Chemotherapeutic – neuropathy	3 (resolved)
Radiation therapy – epiphora and xerostomia	3 (resolved)

## Discussion

Clinical presentation

The clinical features of this neoplasm originating in the nasal cavity are usually caused by local pressure effects on the neurovascular and muscular structures. The commonly observed symptoms pertain to the olfactory, orbital, and otologic systems and include anosmia, parosmia, nasal obstruction, mass descent per nostril, epistaxis, facial pain, epiphora, proptosis, gaze palsy, amaurosis, disorders of refraction, and visual acuity. Neurological manifestations include cranial nerve palsy, especially trigeminal and facial nerves, and headache, which can progress to seizures and meningism if intracranial extension is present. Syndrome of inappropriate antidiuretic hormone secretion with dilutional hyponatremia and Cushing’s syndrome are associated paraneoplastic syndromes reported in the literature. Metastasis to the nodes, usually involving level 2, followed by levels 1, 3, and 7a, can present as a neck swelling. Other common sites of metastasis include the lung, bones, liver, mediastinum, and parotid glands. Differential diagnoses include sinonasal lymphomas, sinonasal undifferentiated carcinomas, and neuroendocrine sinonasal carcinoma [4-6].

Etiology and genetics

Although etiological factors have not been clearly defined, animal studies have observed a link with nitrosamine exposure and viral causes such as polyomavirus, feline leukemia virus, and adenovirus. Some case studies have reported prior exposure to radiation as a possible risk factor, with an average interval of 17 years between exposure and the onset of malignancy [1,6].

Pathology

The cell of origin of ENBs is unclear and assumed to be one of Jacobson’s organs, sphenopalatine ganglion, autonomic ganglia, neural crest cells, or olfactory placodes. Histologically, a well-differentiated ENB is consistent with neuroendocrine and small round blue cell tumors, generally with a high mitotic index and nuclear atypia. They are comprised of small, round, and blue cells with round and uniform nuclei, salt and pepper chromatin distribution, and a high nuclear cytoplasmic ratio. They are lobular with rich vascular fibrous stroma and rosettes, with areas of necrosis. They present with Homer Wright, Flexner Wintersteiner, and perivascular pseudorosettes. Immunohistochemistry staining showed positivity for NSE, synaptophysin, chromogranin A, CD56, S100 protein neurofilaments, calretinin, and glial fibrillary acidic protein and was negative for epithelial and lymphoma markers [1,2,4,6,7].

Staging and prognostication

Morita and Kadish staging, Hyams pathological grading, AJCC 8th edition TNM classification of nasal and maxillary tumors, and Dulguerov’s modified TNM staging are some of the available tools to classify and prognosticate ENBs. Kadish et al. and Dulguerov et al. have used anatomical invasion of the surrounding structures to stage ENBs, while Hyam et al. has given a pathological grading based on tissue architecture, pleomorphism, rosettes, mitotic index, necrosis, and calcification [3,4,8-10].

The Kadish and Dulguerov staging do not reflect the prognosis of the disease accurately in many of the previous studies. Higher T stage was associated with greater chances of positive resection margins. A higher Hyams grade suggested a significantly higher probability of distant and nodal metastasis, ultimately leading to decreased overall survival. Lymph nodal metastasis was associated with poor prognosis, as node-negative patients had a better progression-free survival than node-positive patients (64% vs. 29%). The disease is more aggressive in children, in whom skull base surgery and radiotherapy are associated with long-term and permanent complications such as growth impairment and endocrine and neurologic dysfunction [3,4,6,11].

Molecular advances

In a study that performed comprehensive genomic profiling of refractory ENBs, genomic alterations were observed in the following genes: TP53 (17%), PIK3CA, NF1, CDKN2A, CDKN2C (7% each), and PI3K/mTOR. Amplification of RICTOR, FGF10, FGFR4, FLT4, TrkA/B, and PDGFRB were also observed. Chromosomal alterations include gains at 7q, 9p, 13q, 17p13, 17q, 20p/q, 22q, and Xp/q and losses at 1p, 2q, 3p/q, 6q, 9p, 10p/q, 22q, and Xp/q, especially in higher grade tumors. Deletion at chromosome 11 and gain at 1p are indicators of poor prognosis [2,6,12].

Diagnostics

A complete medical history, including comorbidity history, and physical examination are mandatory before treatment. Complete neurologic and ophthalmologic evaluation is recommended. The tumor appears as a polypoidal, fleshy, and soft mass, which can develop into a friable and ulcerated mass with disease progression. Endoscopic examination followed by biopsy, along with fine needle aspiration cytology of suspected neck nodes, is advised. 1-3 mm slice thickness contrast-enhanced CT and MRI are the first-line investigations for evaluation of ENBs, and care should be taken to differentiate them from cribriform plate meningiomas. Bony erosion of adjacent structures by a homogenous mass with necrotic foci is evident on CT. MRI helps in additional soft tissue delineation, confirming meningeal, perineural, and extradural invasion and differentiating it from secretions. 2-(18F)-fluoro-2-deoxy-d-glucose PET/CT is a useful modality to diagnose nodal metastasis in ENB, as it is highly FDG avid and shows strong contrast enhancement [2,6].

Treatment options

Currently, the standard of treatment is multimodal and involves craniofacial resection, radiotherapy, and chemotherapy. With better therapeutic ratio causing reduced acute radiation toxicities such as mucositis and dermatitis, heavy ion radiotherapy and stereotactic body radiotherapy (SBRT) seem like the best options in our armoury. It is expected that MDT discussions before treatment will have better outcomes for the patient [13].

TThere is no fixed standard regimen for ENBs. The most used induction chemotherapy of choice is three cycles of cisplatin (30 mg/m^2^ per day for 3 days or 100 mg/m^2^ on day 1) and etoposide (100 mg/m^2^ on days 1 and 3), while cisplatin is used concurrently with radiation, with an intercycle interval of three weeks. If the patient is cisplatin-ineligible, carboplatin should be the alternative. Other options include cyclophosphamide, vincristine, ifosfamide, and dacarbazine and Adriamycin-based regimens such as CYVADIC (Adriamycin, 50 mg/m^2^ IV on day 1, cyclophosphamide, 500 mg/m^2^ IV on day 1, vincristine, 1 mg/m^2^ IV on days 1 and 5, and dacarbazine (DTIC--imidazole carboxamide), 250 mg/m^2^ IV on days 1 and 5), VIP (etoposide and ifosfamide plus cisplatin), and CADO (cyclophosphamide, doxorubicin, and vincristine with continuous-infusion cisplatin and etoposide). Despite common use, platinum regimens did not provide a survival benefit compared to other regimens. In patients who received chemotherapy, response rates ranged around 68%-82% across multiple regimens, with a five-year overall survival of 78%. In case of early-stage disease, rhinotomy or endoscopic nasal resection or transnasal procedure was preferred, while advanced disease mandated craniofacial resection. R0 resection is recommended. Orbital exenteration may be required in extensive disease. Elective modified radical neck dissection was generally reserved for patients with clinical adenopathy [2,6,14].

Treatment planning required 3 mm-slice planning contrast CT and a pretreatment MRI with T1-gadolinium contrast and fluid-attenuated inversion recovery T2-weighted sequences. For conventionally fractionated IMRT, the GTV, which received 66-70 Gy, was defined as a visible tumor with contrast enhancement, showing infiltration of adjacent structures. The CTV, which was prescribed at a dose of 60-66 Gy, included microscopic tumor extension, with studies recommending a 3-5 mm margin to GTV and editing at anatomical boundaries. In postoperative patients, the tumor bed and ipsilateral nodal levels 1a-5, 7a were included in the CTV. Elective nodal irradiation included contralateral nodal levels 1b-4. Elective nodal irradiation, even in a clinically negative neck, provided improved regional control but did not translate into a survival benefit. PTV margins were given according to institutional protocols, with studies suggesting a 3-5 mm isotropic margin to the CTV. The main organs at risk comprise the entire optic pathway and the orbit, along with the brainstem. Trials with proton and carbon beams have shown a five-year relapse-free survival of 71%, with acceptable late toxicities [2,13-15].

A Surveillance, Epidemiology, and End Results (SEER) database analysis of 511 ENB patients showed a five-year overall survival of 73% for surgery with radiotherapy, 68% for surgery alone, and 35% for radiotherapy alone. Chemotherapy data were not available and not considered [16].

Patients with higher Hyams grade are recommended for treatment intensification. They should receive induction chemotherapy and elective nodal irradiation, even in a clinically node-negative neck, due to the high propensity of nodal metastasis. Review of neck failures estimated the incidence of cervical metastasis from 17% to 33%, and multiple retrospective studies have suggested that a decreased cervical nodal recurrence is achieved with elective nodal RT [11,15,17].

**Figure 3:**
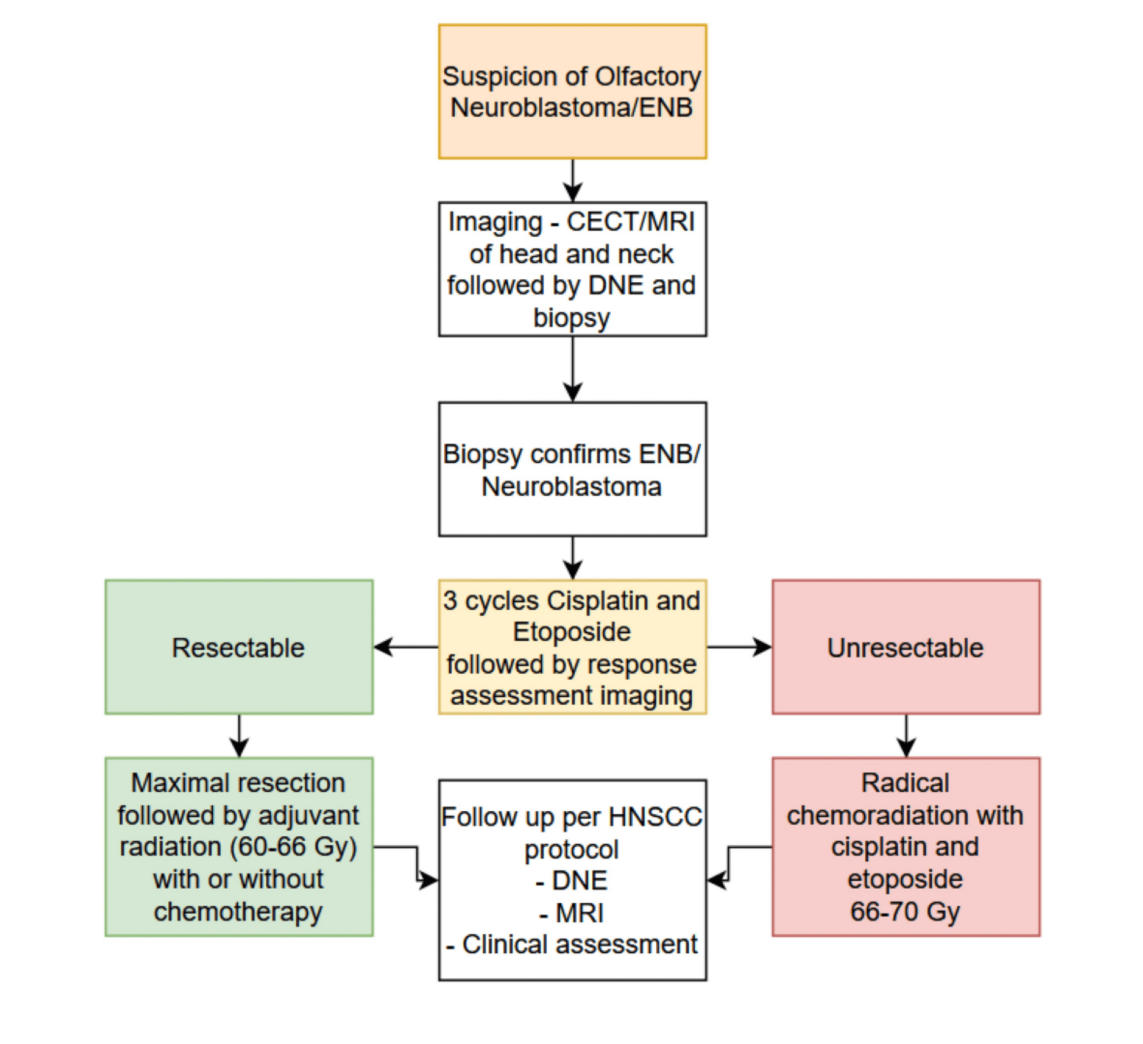
Treatment algorithm for olfactory neuroblastomas/ENBs CECT: Contrast-enhanced computed tomography; DNE: Diagnostic nasal endoscopy; ENB: Esthesioneuroblastoma; Gy: Gray; HNSCC: Head and neck squamous cell carcinoma; MRI: Magnetic resonance imaging

We have prepared an algorithm for diagnostic and therapeutic decision making in Figure 3.

Newer treatment options

In view of improving pathological detection of the tumor and comprehensive molecular and genomic profiling, sporadic case studies and small trials have demonstrated disease response to targeted drugs such as Bcl-2 inhibitors, sunitinib, pazopanib, imatinib, nivolumab, pembrolizumab, vismodegib, everolimus, cobimetinib, trametinib, palbociclib, azacytidine, and decitabine [12].

Follow-up

Acute toxicities of radiation are consistent with those of radiation to the other subsites of head and neck squamous cell carcinoma. Late toxicities are similar to the post-operative complications of head and neck surgeries and irradiation and include xerophthalmia, xerostomia, cataract, parosmia, hearing loss, with some patients rarely experiencing radiation-induced temporal lobe necrosis or optic nerve damage [2,15].

Local recurrences are estimated to be around 30% and regional recurrences 15%-20%, with 30%-50% of the patients amenable to salvage therapy. Distant metastasis accounts for <10% and is associated with poor outcomes. Regular clinical examinations with endoscopy and imaging with MRI should be performed as part of the follow-up protocol, as for HNSCCs. Since a significant proportion of patients are salvageable, a robust follow-up schedule is recommended, with clinical assessment and MRI every four to six months for five years and annually thereafter.

Tumor recurrences can be treated with palliative chemotherapy and SBRT [18], respecting organ constraints and cumulative dose not to incur irreparable toxicity. Median doses of up to 40 Gy in five fractions have been used for reirradiation of recurrent tumors [18].

Limitations of our study include its retrospective design and small sample size, which preclude generalizability. There is a lack of long-term toxicity data due to deficient follow-up patterns among patients treated at referral centers. Due to limited resources, baseline PET-CT and molecular analysis were also not feasible.

## Conclusions

Prognosticating tools have been erratic in the context of ENBs with inhomogeneity of pathological specimen assessment and grading, which could potentially affect treatment. The lack of randomized data due to the rarity of ENBs should be addressed with multicentric collaboration, central pathology and radiology review, and adequate structuring of treatment algorithms. IMRT provides an excellent means of disease control in the radical and relapsed settings. Molecular analysis and targeted therapy may hold the key to extending survival in relapses.
